# Piloting the Feasibility and Preliminary Impact of Adding Birth HIV Polymerase Chain Reaction Testing to the Early Infant Diagnosis Guidelines in Kenya

**DOI:** 10.1097/INF.0000000000003172

**Published:** 2021-05-07

**Authors:** Sarah Finocchario-Kessler, Catherine Wexler, Melinda Brown, Kathy Goggin, Raphael Lwembe, Niaman Nazir, Brad Gautney, Samoel Khamadi, Shadrack Babu, Elizabeth Muchoki, Nicodemus Maosa, Natabhona Mabachi, Yvonne Kamau, May Maloba

**Affiliations:** From the * Department of Family Medicine, University of Kansas Medical Center, Kansas City, Missouri; † Children’s Mercy Kansas City, Health Services and Outcomes Research, Kansas City, Missouri; ‡ School of Medicine, University of Missouri-Kansas City, Kansas City, Missouri; § Center for Virus Research, Kenya Medical Research Institute, Nairobi, Kenya; ¶ Department of Preventive Medicine, University of Kansas Medical Center, Kansas City, Missouri; ∥ Global Health Innovations, Dallas, Texas; ** Global Health Innovations—Kenya, Nairobi, Kenya.

**Keywords:** birth HIV testing, HIV polymerase chain reaction testing, early infant diagnosis, retesting, Kenya

## Abstract

**Background:**

In Kenya, standard early infant diagnosis (EID) with polymerase chain reaction (PCR) testing at 6-week postnatal achieves early treatment initiation (<12 weeks) in <20% of HIV+ infants. Kenya’s new early infant diagnosis guidelines tentatively proposed adding PCR testing at birth, pending results from pilot studies.

**Methods:**

We piloted birth testing at 4 Kenyan hospitals between November 2017 and November 2018. Eligible HIV-exposed infants were offered both point-of-care and PCR HIV testing at birth (window 0 to <4 weeks) and 6 weeks (window 4–12 weeks). We report the: proportion of infants tested at birth, 6-week, and both birth and 6-week testing; median infant age at results; seropositivity and antiretroviral therapy initiation.

**Results:**

Final sample included 624 mother-infant pairs. Mean maternal age was 30.4 years, 73.2% enrolled during antenatal care and 89.9% had hospital deliveries. Among the 590 mother–infants pairs enrolled before 4 weeks postnatal, 452 (76.6%) completed birth testing before 4 weeks, with 360 (79.6%) testing within 2 weeks, and 178 (39.4%) before hospital discharge (0–2 days). Mothers were notified of birth PCR results at a median infant age of 5.4 weeks. Among all 624 enrolled infants, 575 (92.1%) were tested during the 6-week window; 417 (66.8%) received testing at both birth and 6-weeks; and 207 received incomplete testing (93.3% only 1 PCR and 6.7% no PCR). Four infants were diagnosed with HIV, and 3 infants were initiated on antiretroviral therapy early, before 12 weeks of age.

**Conclusions:**

Uptake of PCR testing at birth was high and a majority of infants received repeat testing at 6 weeks of age.

Countries continue to grapple with the challenges of delayed pediatric HIV diagnosis and late antiretroviral therapy (ART) initiation, which contribute to preventable infant and child mortality. In Kenya, infants with HIV initiate ART between 3.7 and 5.9 months of age.^1–4^ This is well after the 8–12 weeks postnatal peak in HIV-related infant mortality^5^ and misses the threshold for early ART initiation (<12 weeks) when infant mortality can be decreased by 76%.^6^

The traditional approach to early infant diagnosis (EID) programs in lower resource settings is laboratory-based HIV polymerase chain reaction (PCR) testing between 4 and 6 weeks postnatal, when PCR sensitivity and specificity are optimal to identify cases of both intrauterine and intrapartum HIV transmission.^7^ In contrast, high-income countries test infants exposed to HIV at birth, to expedite treatment for infants infected in-utero. Such immediate ART initiation has been associated with smaller viral reservoir development^8^ and faster time to viral suppression.^9^ In a pilot study in Lesotho, adding birth HIV PCR testing reduced the median age at ART initiation from 14.8 to 6.4 weeks.^10^ In South Africa, routine birth PCR testing for all infants exposed to HIV <4 weeks of age has increased the proportion of neonates diagnosed with HIV and initiated on ART,^11–13^ with 93% of infants in 1 district initiating ART within the first month of life.^13^ There is some concern, however, that return for subsequent infant testing may be too low. A study in Free State, South Africa found that after the receipt of birth PCR test results, only 44% of uninfected infants returned for a second PCR test at 10 weeks.^14^ Implementation studies are critical to better assess the impact of birth testing on infant outcomes and engagement in the EID continuum of services.

With the introduction of diagnostic strategies for infant point of care (PoC) HIV testing in 2016,^15,16^ Kenya was among the countries which engaged in pilot projects to assess the feasibility and impact of PoC testing for infants exposed to HIV.^17–19^ At the same time, Kenya revised the guidelines for EID to include PCR testing at birth, 6-week, 6 months and 12 months with antibody testing at 18 months.^20^ The recommendation for birth PCR testing was provisional, pending results of pilot projects. To date, birth PCR testing is not yet implemented as standard of care across Kenya. We report findings from a larger pilot study of simultaneous infant HIV testing strategies (PCR and PoC) at birth and 6 weeks of age to assess the feasibility and impact of implementation in Kenya. This paper details findings from the implementation of birth and 6-week HIV testing using laboratory-based PCR.

## METHODS AND MATERIALS

### Study Setting

This pilot study occurred in 4 Kenyan government hospitals with medium to high patient volume (mean of 8–15 infants exposed to HIV per month). Study hospitals were located in varied regions of the country; 1 in the coastal region (urban), 1 in the central region (urban) and 2 in the western region of Kenya (1 urban and 1 peri-urban) with pediatric HIV infection rates ranging from 3.4% to 4.2%.^21^

### Participant Eligibility, Recruitment, Consent

Eligible participants included women living with HIV >18 years of age presenting for care at the study hospitals during (1) pregnancy (antenatal care/prevention of mother to child transmission of HIV [PMTCT] services), (2) delivery or (3) postnatal period (<12 weeks of infant age). Eligible participants were enrolled from June 2017 to November 2018; however, the period of analysis was limited to November 3, 2017 to November 3, 2018 to avoid conflating the impact of the nationwide nursing strike (June 6, 2017 to November 3, 2017) on the pilot results. All eligible candidates were informed about the purpose of the research, potential benefits and risks, and the study procedures by trained research or clinical staff. Participants provided written informed consent before study enrollment. A detailed description of study procedures has been previously published.^17^ The study was approved by the Institutional Review Boards at the Kenya Medical Research Institute (SSC 3390) and the University of Kansas Medical Center.

### Procedures

Infants of mothers living with HIV enrolled in the study were eligible for PCR testing at 2 time points: at birth (0 to <4 weeks) and at 6 weeks of age (4–12 weeks). Those tested within the optimal window for each test were documented: at birth (0–2 weeks) and 6 weeks (4–8 weeks). These time points aligned with HIV PCR testing time targets outlined in the 2016 Kenyan National Guidelines for EID^20^; though, at the time of the pilot study, birth testing was not being routinely implemented. Established standard of care procedures were used for PCR test sample collection and processing: a dried blood spot sample was collected via heel stick and sent by courier to the hospital’s designated central laboratory, processed, and then paper-based results were mailed back to the hospital, where the mother was notified of the results at the next clinical encounter. At both testing time points, if the PCR result was positive, the infant was linked to the comprehensive care center for ART initiation, at which time a confirmatory PCR sample was drawn and processed. Infants continued receiving ART until the confirmatory PCR result determined the infant’s final HIV status.^20^ If the PCR result was negative, mothers were counseled on retesting requirements.

### Measures

The primary outcomes were the proportion of infants who received a birth test and the proportion of infants who returned for a 6-week test. We also distinguished between the optimal window (0–2 weeks for birth testing, and 4–8 weeks for 6-week testing) and the broader window (0 to <4 weeks for birth testing, and 4–12 weeks for 6-week testing) for age-specific infant HIV testing. Retention outcomes included (1) no test received, (2) receipt of birth PCR only, (3) receipt of 6-week PCR only and (4) receipt of birth and 6-week PCR.

Secondary outcomes included turnaround times (TATs) of services (sample processing, result notification, ART initiation), PCR test results (proportion positive, negative, failed/indeterminate), and infant age at PCR testing and ART initiation. Missed opportunities included the number of infants who presented for care during the targeted testing windows but did not receive a PCR test. Missed opportunities for PCR testing were documented when known and summarized.

### Analyses

We used descriptive statistics to characterize participant demographics, and calculated the proportion of infants tested with PCR at birth, 6-week, and birth and 6-week. We calculated the median number of days and interquartile range (IQR) from sample collection to PCR test results and from PCR result to mother notification for tests conducted at birth and at 6-weeks. Lastly, median infant age was calculated for age at sample collection, notification of results, and ART initiation (if indicated) for birth and 6-week testing.

## RESULTS

A total of 670 mother–infant pairs were enrolled during the targeted 1-year period for analyses. Forty-six participants were excluded for the following reasons: 11 mother–infant pairs without a documented maternal date of birth (to confirm eligibility >18 years), 2 with maternal age <18 years at enrollment, 2 presented for testing after 12 weeks of age, and 31 who were discharged early due to transfer/move to another facility (n=11), infant mortality (n=14), maternal mortality (n=1), miscarriage/stillbirth (n=4) or withdrew consent (n=1). Six hundred twenty-four enrolled mother-infant pairs were included in final analyses.

### Participant Characteristics

Mean maternal age was 30.2 years, 72.6% of women were married or lived with their partner, and the majority traveled <60 minutes to reach the hospital for PMTCT services. Nearly three-quarters of the women initiated ART before the current pregnancy, and the majority of women (73.2%) enrolled in the study during antenatal care (vs. at delivery or postnatally), with 89.9% delivering in the hospital, and 54.3% of infants were carried to ≥37 gestational weeks.

**FIGURE 1.**
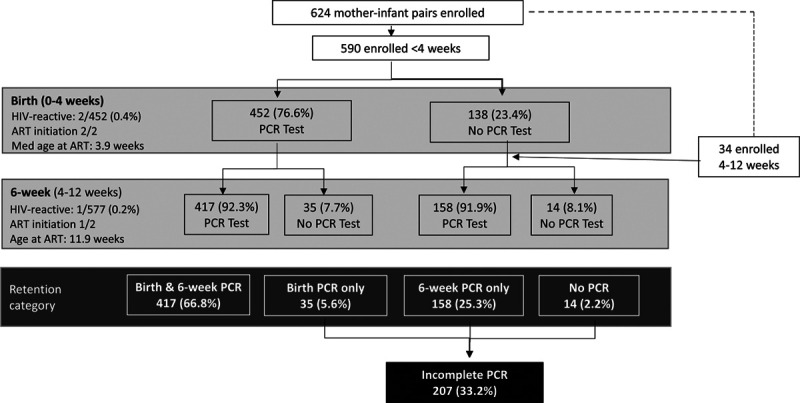
The flow of participants enrolled in the study and the timing of infant HIV PCR testing, timing of HIV diagnosis and ART initiation, and testing retention outcomes: birth PCR only, 6-week PCR only, birth and 6-week PCR or no PCR testing. Those who received only 1 or no test were categorized as receiving incomplete PCR testing.

The timing of infant PCR testing, the proportion diagnosed with HIV, infant age at ART initiation, and the retention categories for testing are outlined in Figure 1.

### Birth Polymerase Chain Reaction Testing

Among the 590 mother–infant pairs enrolled before 4 weeks postnatal, 452 (76.6%) were tested within the birth window of 0–4 weeks of age, and among those 360 of 452 (79.6%) were tested within the optimal window of 0–2 weeks of age. One hundred seventy-eight of 452 (39.4%) were tested before hospital discharge after delivery (0–2 days).

### Six-week Polymerase Chain Reaction Testing

Compared with birth testing, a higher proportion of infants (575 of 624, 92.1%) received testing within the traditional 6-week window, with 546 of 575 (95.0%) tested on time.

### Birth and 6-week Testing

In total, 417/624 (66.8%) received repeat PCR testing (a birth PCR test followed by a repeat PCR test at 6 weeks of infant age). Among these, 332 (79.6%) infants received the birth and 6-week testing sequence within the optimal/on-time windows. The proportion receiving birth and 6-week testing at the 3 urban hospitals averaged 64.0% and was 74.2% at the peri-urban hospital.

### Incomplete Testing

A total of 207 (33.2%) infants exposed to HIV received incomplete PCR testing for the following reasons: 35 (5.6%) birth test only (tested at birth, but not retested at 6 weeks), 158 (25.3%) 6-week test only and 14 (2.2%) with no PCR at either time point.

### Efficiency of Laboratory-based Polymerase Chain Reaction Testing, Results Notification and Antiretroviral Therapy Initiation

Of the 452 infants who received a birth PCR test, there were 448 (99.1%) valid test results (446 negatives and 2 positives) posted to facilities with a median TAT of 14 days (IQR 7–22). Four hundred forty-one (98.4%) mothers were notified after a median time of 3 days (IQR 0–21) of test result.

Of the 575 infants who received a 6-week PCR test, there were 573 valid test results (571 negatives and 2 positives) posted to facilities with a median TAT of 13.5 days (IQR 7–21) from sample collection to return of result. Five hundred sixty-two (98.1%) mothers were notified of their infant’s results after a median of 5 days (IQR 0–20). The median therapeutic TAT of results (sample collection to notification of caregiver) was the same for infants tested at birth (27 days, IQR 14–37) and 6-week (27 days, IQR 12.75–32). Median infant age at birth PCR sample collection was 0.8 weeks (IQR 0.14–1.9) and mothers were notified of results at a median infant age of 5.4 weeks (IQR 2.6–6.3). Both infants diagnosed with HIV at birth were initiated on ART at 3.9 weeks. Among infants tested at 6 weeks, the median infant age at sample collection was 6.1 weeks (IQR 6.0–6.4), mothers were notified by 10.0 weeks (IQR 8.0–11.0), with ART initiation of 1 infant at 11.9 weeks. Infants initiating ART were diagnosed by PCR only; either due to a PoC machine error or provider hesitancy to initiate ART based on PoC results alone. One infant diagnosed by 6-week PCR was not initiated on ART. The mother was notified of the results, but did not return to the hospital, despite repeated efforts and a home visit by a community health worker.

### Missed Opportunities

Of the 172 infants who did not receive a birth PCR test, 34 were enrolled after the birth test window (≥4 weeks of age). Of the remaining 138, 19 (13.7%) presented for testing but only received a birth PoC test, thus reflecting a missed opportunity to provide birth PCR. Approximately 20% (119 of 590) of those enrolled antenatally or at delivery did not return and present for EID early enough to be tested within the birth window.

Of the 49 of 624 (7.8%) infants who did not receive PCR testing at the well-established 6-week period, only 1 infant showed a documented hospital visit at 6-week, reflecting a missed opportunity to provide PCR testing at the same time. Among those not tested at 6 weeks, 71.4% (35 of 49) had received a PCR test during the birth window; only 2.2% of enrolled infants did not receive any PCR test during the first 12 weeks of life.

## DISCUSSION

These promising data demonstrate that earlier and repeated testing within the first 2 months of life is feasible with the established PCR laboratory infrastructure in Kenya. Nearly three-quarters of mother–infant pairs received testing during the birth windows. South Africa’s high coverage of birth HIV testing after nationwide implementation,^12,13^ holds promise for relatively rapid uptake in similar settings. Similar to the experience in Eswatini,^22^ this pilot study in Kenya did not observe the concern that fewer infants tested at birth may return for retesting; with nearly all (92.3%) infants tested at birth returning for a follow-up 6-week PCR test. A similarly high proportion of infants who were not tested at birth received a 6-week test (91.9%), indicating the well-established protocol of 6-week infant testing. In total, 66.8% of the sample achieved repeat testing (birth and 6-week). Only 2.2% of infants did not return for testing at either time point.

Formative qualitative interviews with parents and HIV care providers conducted before implementation revealed that some parents feared an infant’s positive diagnosis at birth could hinder mother–infant bonding and thus preferred traditional 6-week testing.^23^ Thus, continuous and comprehensive counseling throughout pregnancy is necessary to shape women’s expectations for early infant testing within the first 2 weeks of life and prepare women for the complete EID cascade of care. While 0–2 weeks is the optimal target for birth testing, we included a more generous range of 0–4 weeks. This allowed a more flexible window for infants delivered at home, and with an average TAT of 10 days for PCR test results in Kenya,^21^ infants tested <4 weeks could still have PCR results available by the 6-week retest (median infant age at the notification of birth results in the pilot was 5.4 weeks). If test and treat guidelines are followed for birth testing, positive infants could be diagnosed and initiated on ART by their 6-week visit, achieving targets for early ART initiation. Among the 3 infants initiated on ART in our study, 2 were diagnosed during the birth window and both initiated within the first month of life, and the infant diagnosed at 6-week was initiated on ART before the 12-week target. Other studies evaluating birth PCR testing found age at ART initiation ranged from 9 days to 9.7 weeks,^10,24–26^ while studies evaluating at birth PoC testing found age at ART initiated ranged from 2 days to approximately 1 month of age.^22,25^

Given the success in early ART initiation that many birth PCR programs have achieved, the consistent TAT of 2 weeks for PCR results in Kenya, and the challenges to implementing and sustaining birth PoC testing (cost, cartridge stock outs, machine errors, provider training, secure machine storage, reduced sensitivity at birth),^23,27,28^ it may be more pragmatic at this time for policy makers to add the more familiar PCR test at birth, rather than investing in the infrastructure and training required for birth PoC testing. In our parallel pilot study of PoC testing at these same sites, we experienced higher rates of missed testing opportunities due to machine/stock outs or invalid results (23.3%),^29^ and delays in ART initiation still occurred largely due to the initial novelty of PoC in this setting.^29,30^ Other larger PoC studies have reported more favorable findings,^19,31^ and the benefit of PoC may be greater in settings with longer delays for laboratory-based PCR results. Furthermore, PCR HIV testing at birth and 6-week has been assessed as cost-effective, compared with EID at 6 weeks alone.^32^ Thus, the addition of birth PCR may be more feasible for nationalwide implementation in some countries.

To ensure hospital readiness and maximize benefits, barriers to implementation of birth testing must be addressed. One very practical, system-level barrier identified in a parallel study with repeated focus groups with providers over the course of the birth PCR implementation period, was that infant IDs are not usually designated until a first postnatal visit after 2 weeks; thus, confusion around infant IDs for those tested in maternity before discharge hindered early implementation.^33^ Furthermore, the practice of delivering in a hospital but seeking infant postnatal services in a closer health facility, could complicate the ability to accurately document repeat testing. Thus, history of birth testing will be important for providers to assess when infants present for routine EID at 6 weeks.

The critical gains in neonatal diagnosis and ART initiation achieved by birth testing can be lost without strong systems to support pediatric ART adherence, viral loading monitoring, and retention in care, as cautioned by researchers in South Africa.,^11,13,14^ Thus, after the initial birth test, structured interventions are needed to support the completion of EID services and quality of pediatric HIV management,^34^ recognizing that key patient-level psychosocial (HIV stigma and nondisclosure), financial, and logistical (childcare and transportation) barriers to EID retention remain.^35–38^

## LIMITATIONS

This pilot study has several limitations to consider. The sample size was relatively small with only 4 government hospitals included for a duration of 1 year. Given the advances in antenatal coverage of PMTCT services, the proportion of infants diagnosed with HIV were few (<1%). This limited the generalizability of findings specific to infants diagnosed with HIV, including the efficiency and infant age of ART initiation, which are priority outcomes when considering varied infant testing strategies. The findings specific to infants who were not diagnosed with HIV, which reflect the vast majority of infants enrolled in EID in Kenya, demonstrate the feasibility of birth PCR testing with actionable test results by the time repeat testing is due at 6 weeks of age. We also acknowledge that the wide window afforded for “6-week” testing in this study (4–12 weeks), exceeds the target of EID before 8 weeks of age. Our ability to accurately characterize missed opportunities for birth PCR testing was limited by incomplete documentation that failed to specify a reason for the lack of testing, thus we could not distinguish between cases in which the infant never presented for HIV testing before 4 weeks or the provider failed to request an early test during the visit. Lastly, these findings are limited to women seeking facility-based PMTCT and EID services in Kenya and the pilot study did not include a comprehensive cost-effectiveness analysis.

## CONCLUSIONS

Our data suggest that simply accelerating the timeline for infant HIV testing with traditional laboratory-based PCR testing at birth can reduce infant age at HIV diagnosis and does not hinder 6-week retesting, which remains necessary to diagnose early postnatal HIV transmission.

## ACKNOWLEDGMENTS

The Kenya Medical Research Institute, Global Health Innovations, and Children’s Mercy Kansas City were collaborative partners in these efforts. We are grateful to the mother–infant pairs and hospital staff who participated in the study. We also acknowledge the critical role of our government partners at the Kenya National AIDS and STI Control Program (NASCOP) and County Ministers of Health. We thank the Director of KEMRI for permission to publish this manuscript.
